# From Ecological Niche to Conservation Planning; Climate‐Driven Range Dynamics of *Ephedra intermedia* in Central Asia

**DOI:** 10.1002/ece3.71127

**Published:** 2025-03-16

**Authors:** Muhammad Waheed, Fahim Arshad, Sehrish Sadia, Beatrice Ambo Fonge, Abeer Al‐Andal, Asma Jabeen, Shalom Dilshad

**Affiliations:** ^1^ Department of Botany University of Okara Okara Pakistan; ^2^ Department of Biological Sciences University of Veterinary and Animal Sciences Pattoki Pakistan; ^3^ Department of Plant Science University of Buea Buea Cameroon; ^4^ Department of Biology, College of Science King Khalid University Abha Saudi Arabia; ^5^ Department of Environmental Sciences Fatima Jinnah Women University Rawalpindi Pakistan

**Keywords:** climate change impact, conservation planning, ecological niche modeling, *Ephedra intermedia*, habitat suitability

## Abstract

*Ephedra intermedia*, a medicinally significant plant, is an important component of arid and semi‐arid ecosystems across Central and South Asia. This research sought to predict the present and future distribution of *E. intermedia* by applying ecological niche modeling (ENM) methods. The model incorporated comprehensive bioclimatic and edaphic variables to predict the species' habitat suitability. The results demonstrated high predictive accuracy, highlighting the importance of temperature seasonality, annual temperature range, soil pH, and nitrogen content as key species distribution determinants. The current habitat suitability map revealed core areas in Afghanistan, Pakistan, and Tajikistan mountain regions. Under future climate change scenarios (SSP2‐4.5 and SSP5‐8.5) for the 2050s and 2070s, the model projected a significant upward and northward shift in suitable habitats, coupled with a notable contraction in the extent of highly suitable areas, particularly under the high‐emission SSP5‐8.5 scenario. The predicted range shifts reflect the species' sensitivity to increasing temperatures and changing precipitation patterns. This suggests a potential loss of suitable habitats in low‐elevation and southern parts of its range. Including edaphic factors in the model provided novel insights, specifically highlighting the critical role of soil properties, such as soil pH and nitrogen content, in shaping the ecological niche of *E. intermedia* . These findings complement the observed upward and northward shifts in habitat suitability under future climate scenarios, emphasizing the species' reliance on high‐altitude refugia as climate conditions change. The results underscore important implications for conservation planning, suggesting that strategies should prioritize the protection of these refugial habitats while also considering measures such as habitat connectivity and assisted migration to support the species' adaptation to shifting environmental conditions.

## Graphical abstract

This study predicts the current and future distribution of Ephedra intermedia using ecological niche modeling, incorporating bioclimatic and edaphic variables. Results indicate temperature seasonality, annual temperature range, soil pH, and nitrogen content as key distribution determinants, with significant range shifts projected under future climate scenarios. The findings highlight the need for conservation strategies prioritizing high‐altitude refugia and adaptive measures to safeguard this ecologically and medicinally important species.

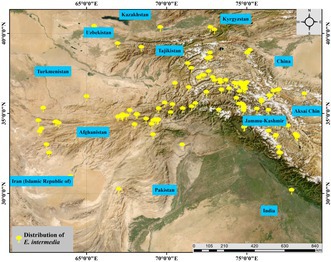

## 1 Introduction

Climate change is one of the most pressing global environmental challenges, significantly affecting biodiversity and ecosystem stability (Abbass et al. 2022). The rapid alterations in temperature and precipitation patterns profoundly impact species distribution, particularly for those adapted to specific climatic and edaphic conditions (Dong et al. 2022). With increasing global temperatures and more erratic weather patterns, species must adapt, relocate, or risk local extinction (Pigot et al. 2023). These changes are expected to be more pronounced for plant species with narrow ecological niches, such as those adapted to arid and semi‐arid environments (Zhao et al. 2024). Understanding how these species respond to changing climatic conditions is crucial for predicting future biodiversity dynamics and developing effective conservation strategies (Waldvogel et al. 2020; Trew and Maclean 2021). Edaphic variables are soil‐related factors that influence the distribution, growth, and ecological interactions of plant species (Waheed et al. 2022; Sadia et al. 2024). These variables play a critical role in shaping ecological niches, particularly in environments where soil characteristics are primary determinants of habitat suitability (Arshad, Haq, et al. 2024; Arshad, Shoaib, et al. 2024).

Ecological niche modeling (ENM) has emerged as a powerful tool for predicting species distributions and assessing potential range shifts under future climate scenarios (Melo‐Merino et al. 2020). ENM uses species occurrence data and environmental variables to estimate a species' niche and project its geographic distribution across different climatic conditions (Pshegusov et al. 2022). Among various modeling techniques, MaxEnt (Maximum Entropy) is widely recognized for its robustness and predictive accuracy, especially when dealing with presence‐only data (Phillips et al. 2006). By integrating bioclimatic predictors, ENM provides insights into the factors that influence species distributions and helps forecast shifts in suitable habitats in response to climate change (Rather et al. 2022). Such predictive models are invaluable for identifying at‐risk species and guiding conservation planning (Swan et al. 2021; Bai et al. 2024). Climate change is already causing observable range shifts across multiple taxa, with numerous species migrating towards higher altitudes or latitudes in search of suitable habitats (Spence and Tingley 2020). Such shifts may result in altered community dynamics, habitat loss, and intensified competition, posing significant threats to species that cannot relocate or adapt rapidly enough (Vitasse et al. 2021). For species with specialized habitat requirements, like many arid‐adapted plants, predicting potential range contractions and identifying climate refugia are critical steps in mitigating the impacts of climate change (Buckner and Danforth 2022). ENM offers a strategic approach to anticipate these changes and inform adaptive conservation measures, ensuring the long‐term preservation of vulnerable species and their habitats (Zurell et al. 2022).

*Ephedra intermedia* Schrenk & C.A. Mey is a plant species widely recognized for its adaptive traits, therapeutic properties, and significant role in traditional medicine systems (Guo, Gao, et al. 2023; Guo, He, et al. 2023). The species thrives in arid and semi‐arid environments and contributes to ecosystem stability through various pathways. Although not explicitly identified as a keystone or foundation species, *E. intermedia* plays a critical ecological role by preventing soil erosion, stabilizing desert environments, and providing structural support that facilitates microhabitats for other species, such as soil‐dwelling organisms and small fauna (Minami et al. 2021; Zhang et al. 2024). These roles are particularly vital in ecosystems that experience higher levels of abiotic stress, such as extreme temperatures, low precipitation, and nutrient‐poor soils. Despite its ecological and medicinal importance, the current and future ecological niche of *E. intermedia* remains uncertain, particularly under the influence of climate change. The study aims to assess the current ecological suitability of *E. intermedia* in Central Asia, identify key environmental variables influencing its habitat, and project future distribution patterns under climate change scenarios for the 2050s and 2070s. Climate change significantly impacts species distribution, forcing plants to adapt, shift their ranges, or face local extinction, especially in environments with distinct climatic constraints (Hulme 2005; Spence and Tingley 2020). Studies have shown that many montane and drought‐adapted species migrate towards higher elevations and latitudes in response to rising temperatures (Lenoir et al. 2008; Lindner et al. 2010; Zu et al. 2021), a trend observed in *Ephedra sinica* and *Ephedra distachya* (He et al. 2021; Zhang et al. 2022, 2024). It is hypothesized that *E. intermedia* will exhibit upward and northward range shifts in response to increasing temperatures and altered precipitation patterns, with core habitats in low‐elevation and southern regions contracting and new suitable areas emerging at higher elevations and latitudes. The findings offer valuable understanding of the ecological requirements of *E. intermedia* , guiding the development of effective conservation strategies in the face of rapid environmental change.

## 2 Methodology

### 2.1 Ephedra intermedia

**FIGURE 1:**
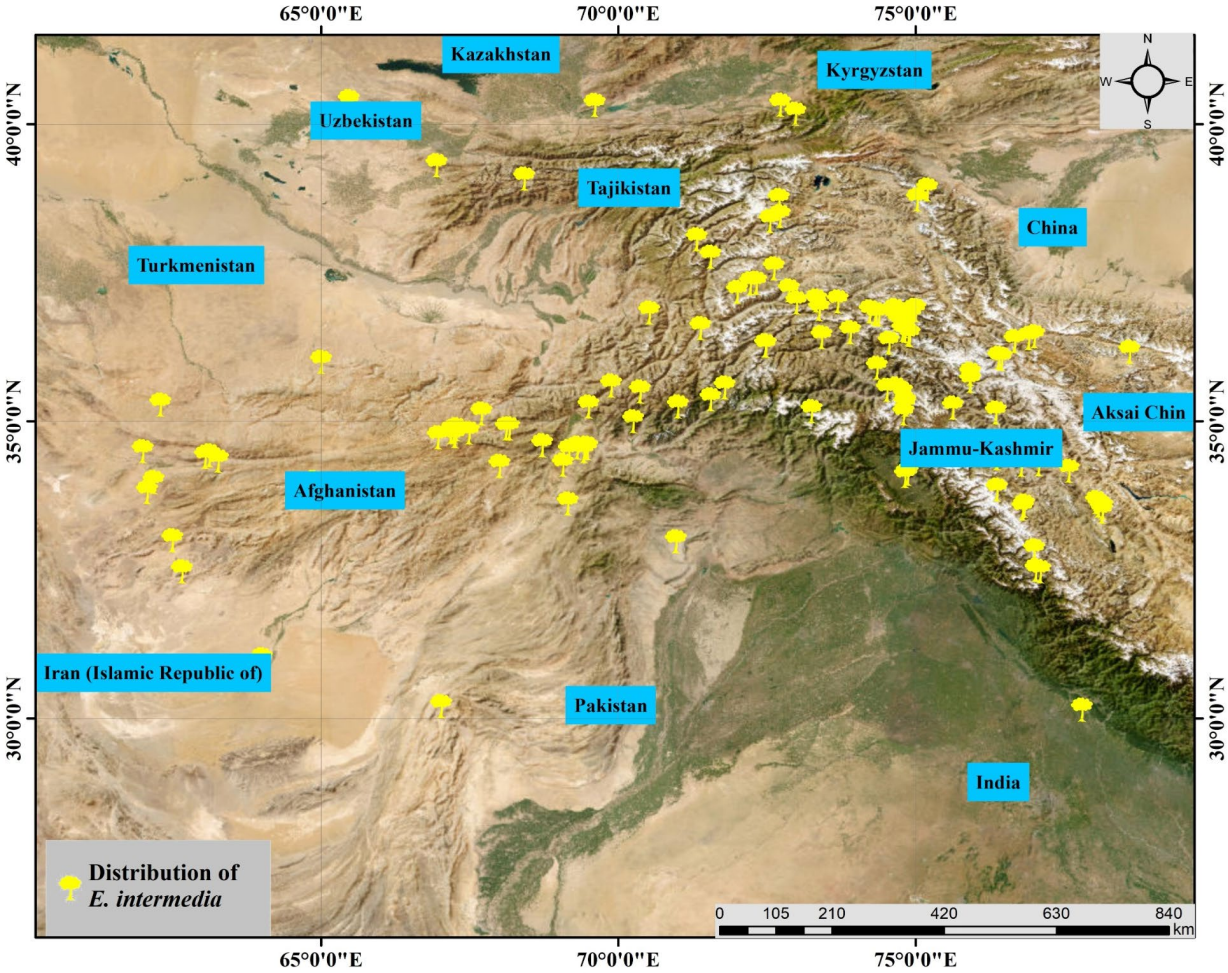
Distribution map of *E. intermedia* based on occurrence records from the Global Biodiversity Information Facility (GBIF) and literature. The map highlights the widespread but fragmented distribution of the species across Central Asia, including areas in China, Afghanistan, Kazakhstan, Pakistan, and Tajikistan.

*Ephedra intermedia* is a dioecious shrub, typically reaching up to 1 m in height, characterized by its jointed, green stems and small, scale‐like leaves (González‐Juárez et al. 2020). The plant thrives in arid and semi‐arid environments, commonly found in deserts, grasslands, floodplains, river valleys, slopes, cliffs, and sandy beaches at elevations ranging from 100 to 4000 m (Gul et al. 2017). Its distribution spans across Central and East Asia, including regions of China, Kazakhstan, Afghanistan, and Pakistan (Figure 1). *Ephedra intermedia* plays a vital role in stabilizing desert ecosystems and preventing soil erosion (Liu et al. 2020). However, overexploitation for its medicinal properties, particularly for ephedrine extraction, has led to concerns about population decline (Tokuda et al. 2024; Fan et al. 2024). Conservation efforts are essential to ensure the sustainability of this species, considering its ecological significance and medicinal value.

## 3 Data Collection and Screening

In this study, we gathered a detailed dataset to predict the distribution of *E. intermedia* . The occurrence dataset comprised 190 records: 170 data points were sourced from the GBIF (www.gbif.org), whereas 20 additional records were obtained from published literature (Chaudhri 1957; Kakiuchi et al. 2007; Jan et al. 2009; Nowak et al. 2016; Khan et al. 2018; Hayashi et al. 2019; Basit et al. 2021; Li et al. 2024) (Table S1). To ensure data quality, duplicate records were removed, and occurrence points outside the known range of *E. intermedia* were excluded. To minimize spatial autocorrelation, we applied a spatial thinning process at a 1 km resolution. The cleaned occurrence dataset was then projected onto a 2.5 arc‐minute grid for spatial consistency with the environmental predictors. We included 19 bioclimatic variables with a spatial resolution of 2.5 arc minutes, retrieved from WorldClim version 2.1 (www.worldclim.org, accessed March 20, 2022). Furthermore, we incorporated 10 edaphic variables from SoilGrids (https://soilgrids.org/, accessed March 20, 2022) to improve the model's ecological representation (Table S2). High‐resolution elevation data (30 arc seconds) were processed using ArcGIS 10.5 to derive topographic features such as slope and aspect, which were subsequently included as predictive variables. To assess potential shifts in habitat suitability under future climate scenarios, we utilized two Shared Socioeconomic Pathways (SSPs): SSP2‐4.5, indicating a moderate emissions pathway, and SSP5‐8.5, representing a high‐emissions scenario. These projections were based on climate data from the Coupled Model Intercomparison Project Phase 6 (CMIP6) for the time frames 2050s (2041–2060) and 2070s (2061–2080). We selected the BCC‐CSM2‐MR global climate model for its high spatial resolution and reliable performance in simulating bioclimatic variables.

## 4 Initial Processing of Environmental Variables

**FIGURE 2:**
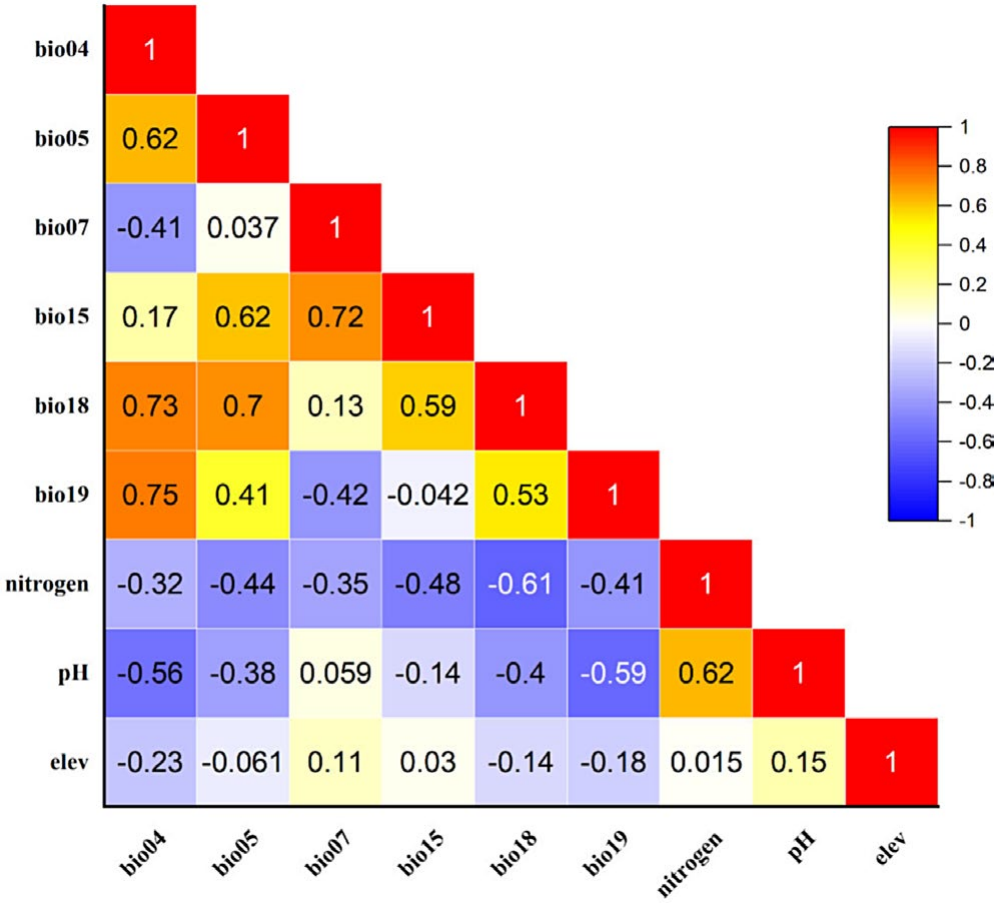
Pearson correlation analysis generated a heatmap illustrating the pairwise correlations between the climatic and biophysical variables used in the distribution modeling of *E. intermedia* , with a correlation threshold set at ±0.8.

A two‐step procedure was employed to ensure the selection of independent and ecologically relevant variables. First, Pearson correlation analysis was conducted to detect multicollinearity among the environmental variables. Variables with a correlation coefficient greater than 0.8 were excluded to minimize redundancy and multicollinearity issues (Graham 2003). Following this, a subset of nine variables was identified based on their ecological significance and minimal inter‐correlation: temperature seasonality (bio04), maximum temperature of the warmest month (bio05), temperature annual range (bio07), precipitation seasonality (bio15), precipitation of the warmest quarter (bio18), precipitation of the coldest quarter (bio19), elevation, soil nitrogen, and soil pH (Figure 2).

## 5 Model Calibration and Fine‐Tuning and Validation

MaxEnt software (version 3.4.4) was utilized for ENM of *E. intermedia* (Phillips et al. 2006). To enhance model performance and diminish the risk of overfitting, we tested diverse combinations of regularization multipliers (RM) and feature classes (FC) (Elith et al. 2006, 2011; Phillips et al. 2009). We specifically evaluated eight RM values, ranging from 0.5 to 4 in increments of 0.5, along with six FC (L, LQ, H, LQH, LQHP, and LQHPT) (Anderson and Gonzalez Jr. 2011; Bao et al. 2022; Fourcade et al. 2014; Merow et al. 2013). The ENMeval package in R was applied to optimize parameter selection and minimize overfitting (Kass et al. 2021). The model configurations included the use of a complementary log–log (clog–log) output format, 10‐fold cross‐validation, response curve analysis, and jackknife tests to appraise the status of each environmental predictor (Phillips and Dudík 2008; Peterson et al. 2007).

MaxEnt outputs were exported to ArcGIS 10.5 for spatial analysis. The continuous habitat suitability maps were classified into five distinct categories based on logistic output values: unsuitable (US), low suitability (LS), moderate suitability (MS), high suitability (HS), and very high suitability (VHS). This classification allowed for a consistent evaluation of habitat quality and facilitated the identification of core distribution zones for *E. intermedia* . The accuracy of the optimized ENM was determined using the area under the curve (AUC) of the receiver operating characteristic (ROC) curve (Elith et al. 2011). An AUC score of 0.9 or higher indicated excellent model accuracy (Summers et al. 2012; Yang et al. 2013). Additional evaluation metrics, including the 10% training omission rate (OR10) and the difference between training and testing AUC (AUC.DIFF), were used to assess the model's robustness and potential overfitting (Bai et al. 2018). The future climate projections were analyzed to predict range shifts and changes in habitat suitability for *E. intermedia* during the 2050s and 2070s.

## 6 Results

**FIGURE 3:**
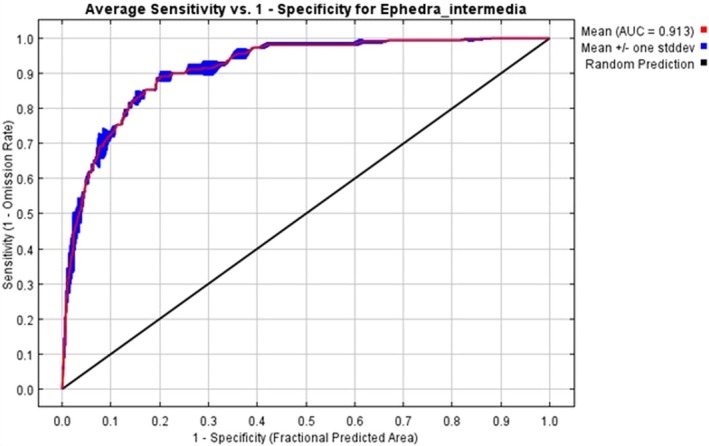
Receiver operating characteristic (ROC) curve for the ecological niche model of *E. intermedia* . The curve illustrates the sensitivity (true positive rate) versus 1‐specificity (false positive rate) across varying threshold levels.

The ENM for *E. intermedia* demonstrated strong predictive performance, as evidenced by the Area Under the ROC Curve (AUC). The mean AUC value was 0.913, indicating a high level of model accuracy (Figure 3). The ROC curve shows a steep rise with minimal false positive rates, further emphasizing the model's robustness. The mean predicted omission closely aligns with the expected omission across different thresholds.

**FIGURE 4:**
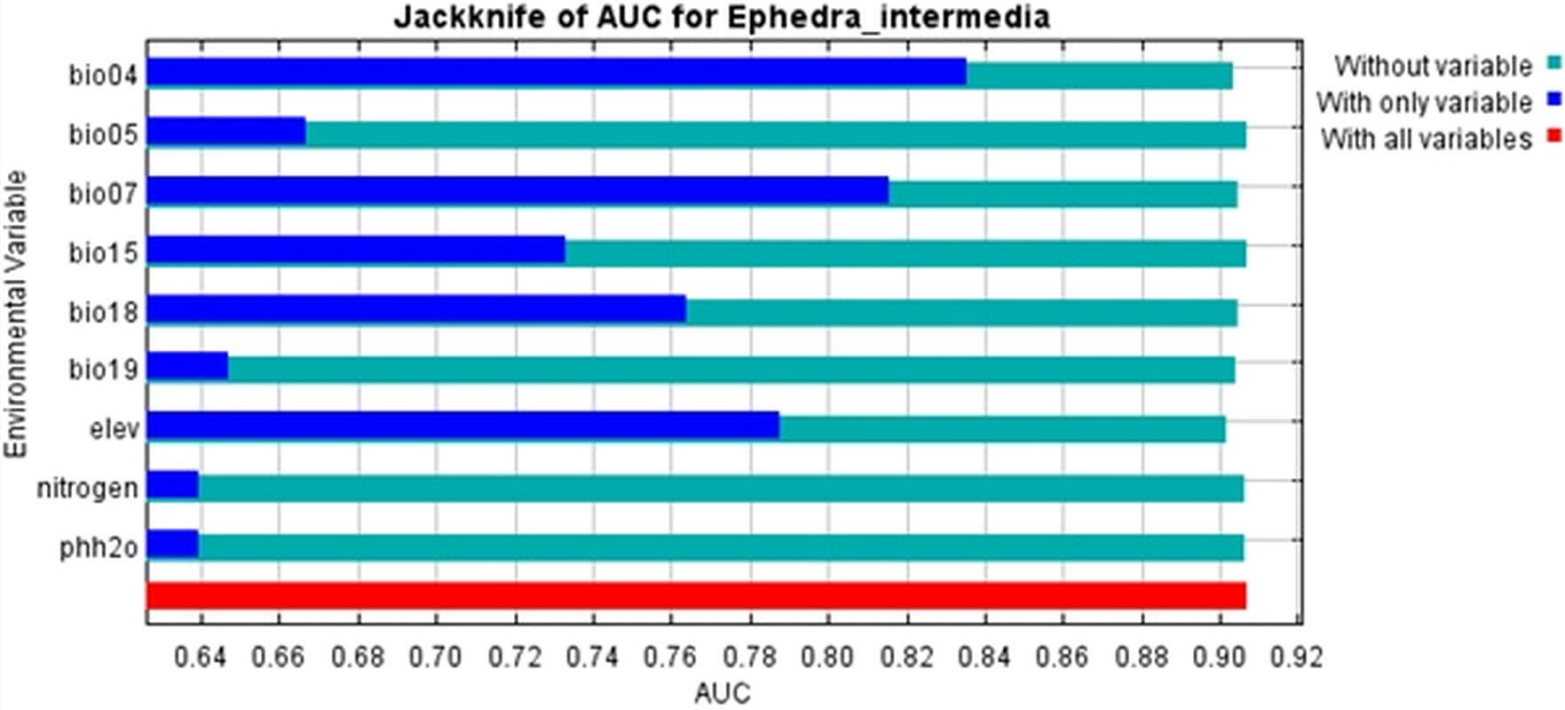
Jackknife analysis of variable importance for the ecological niche model of *E. intermedia* . Blue bars signify the gain or AUC when the variable is excluded, indicating its unique contribution to the model. Green bars indicate the performance when only the specific variable is used, showing its individual predictive power. Red bars indicate the overall model performance with all variables included.

Jackknife tests for variable importance provided insights into the contribution of each predictor variable to the model (Figure 4). Among the environmental variables, soil pH exhibited the highest contribution, resulting in the highest training gain when used alone and a significant drop in training gain when omitted. This suggests that soil pH plays a critical role in determining the suitable habitat for *E. intermedia* . Other important predictors include nitrogen content, elevation, and bioclimatic variables such as bio07 (temperature annual range) and bio15 (precipitation seasonality), all of which contributed substantially to the model gain. The jackknife test for AUC further supports the significance of these variables, as phh2o, nitrogen, and elevation showed the highest AUC values when used in isolation.

## 7 Response of *E. intermedia* to Environmental Variables

**FIGURE 5:**
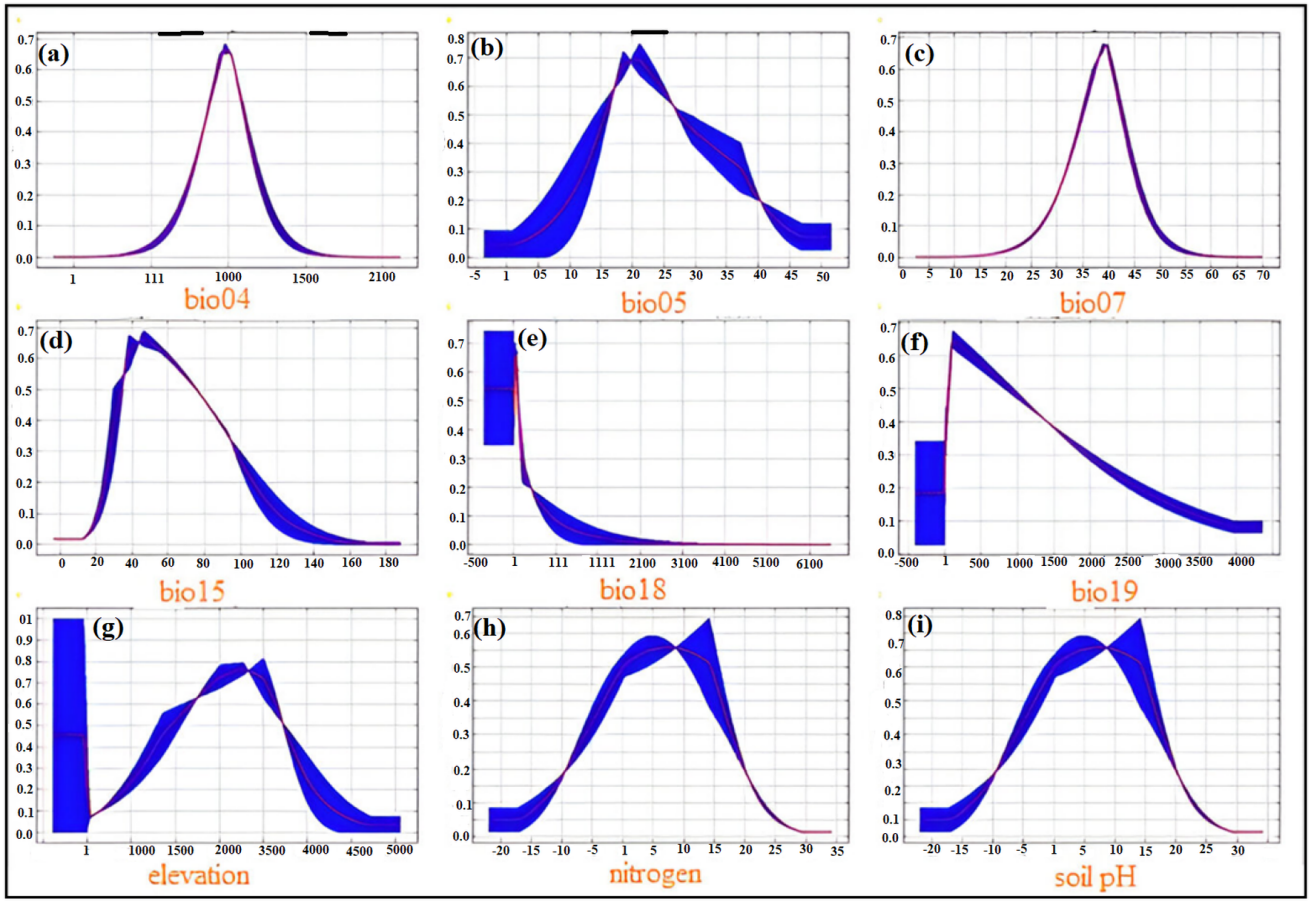
Response curves of *E. intermedia* to key environmental variables. The panels illustrate the species' predicted habitat suitability as a function of (A) bio04, (B) bio05, (C) bio07, (D) bio15, (E) bio18, (F) bio19, (G) elevation, (H) soil nitrogen content, and (I) soil pH (phh2o). The x‐axis represents the value of each environmental variable, while the y‐axis shows the logistic output, indicating habitat suitability. The response curves highlight the ecological preferences of *E. intermedia* .

The response curves for *E. intermedia* indicate the preferences and tolerances for a range of bioclimatic and edaphic factors. For bio04, the logistic output peaks at moderate seasonality values (~1000), suggesting that the species prefers environments with stable yet moderately fluctuating temperatures (Figure 5). The response to bio05 reveals an optimal range between 15°C and 25°C, beyond which the predicted suitability declines sharply, indicating sensitivity to extreme high temperatures. The response to bio07 displays a predilection for areas with an annual temperature range of 35°C to 45°C, highlighting the species' adaptation to regions with significant temperature variability. Similarly, bio15 demonstrates a peak in suitability at moderate precipitation seasonality values (40–60), suggesting that *E. intermedia* favors environments with balanced rainfall distribution throughout the year (Figure 5). Bio18 and bio19 both show a declining response at higher precipitation levels, indicating that *E. intermedia* prefers drier conditions during both the warmest and coldest periods of the year. Regarding edaphic and topographic factors, the response to elevation indicates a bimodal distribution, with optimal suitability observed at mid‐elevations (2000–3500 m). The response to soil nitrogen content highlights an optimal range of 10–15 units, while the suitability decreases outside this range, indicating a preference for moderately fertile soils. The response to soil pH shows a peak at pH values between 5 and 15, suggesting that *E. intermedia* thrives in slightly alkaline to moderately acidic soils. the environmental response curves highlight the adaptability of *E. intermedia* to specific climatic and edaphic conditions, with clear preferences for moderate temperature seasonality, balanced precipitation, mid‐elevation zones, and moderately fertile, slightly alkaline soils.

## 8 Spatial Distribution and Habitat Suitability

**FIGURE 6:**
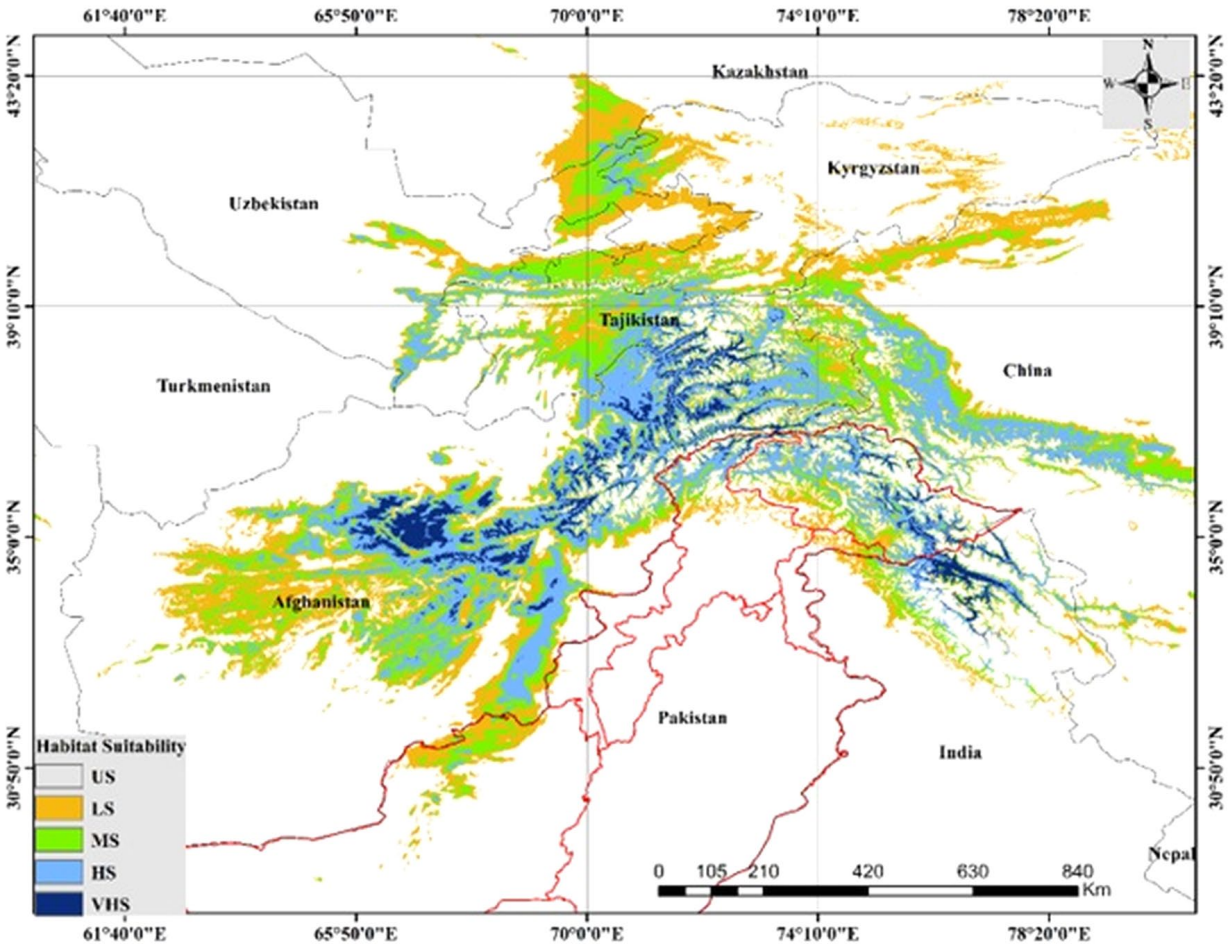
Current predicted distribution map of *E. intermedia* across Central and South Asia. Habitat suitability is categorized into unsuitable (US, gray), low suitability (LS, yellow), moderate suitability (MS, green), high suitability (HS, light blue), and very high suitability (VHS, dark blue).

The predicted habitat suitability map for *E. intermedia* shows its potential distribution across Central and South Asia, categorized into five classes (Figure 6). The spatial analysis highlights several key findings: Regions of VHS are located in the mountainous areas of Afghanistan, Tajikistan, and northern Pakistan, particularly along the Hindu Kush and Karakoram ranges. These areas correspond to the known altitudinal preferences of *E. intermedia* , aligning with the mid‐elevation zones identified as favorable in the response curves. The model also identifies significant areas of MS to HS extending into Uzbekistan, Turkmenistan, and parts of Kyrgyzstan. LS and US habitats are predominantly located in the arid lowlands of Turkmenistan and southern Uzbekistan, as well as the densely vegetated or high‐rainfall areas in northern India and Nepal. The distribution map highlights the transboundary occurrence of *E. intermedia* , indicating potential cross‐border conservation areas, particularly in the shared mountainous regions between Afghanistan, Pakistan, and Tajikistan. *Ephedra intermedia* exhibits HS in key regions of Central and South Asia. In Afghanistan, the Koh‐i‐Baba range and Hindu Kush mountains stand out, while in Pakistan, the Karakoram Range, Swat Valley, and Chitral region are critical. Tajikistan's Pamir‐Alay and Gissar ranges, Kyrgyzstan's Tien Shan mountains, and Uzbekistan's Nuratau Mountains also host significant suitable habitats.

## 9 Projected Future Distribution

**FIGURE 7:**
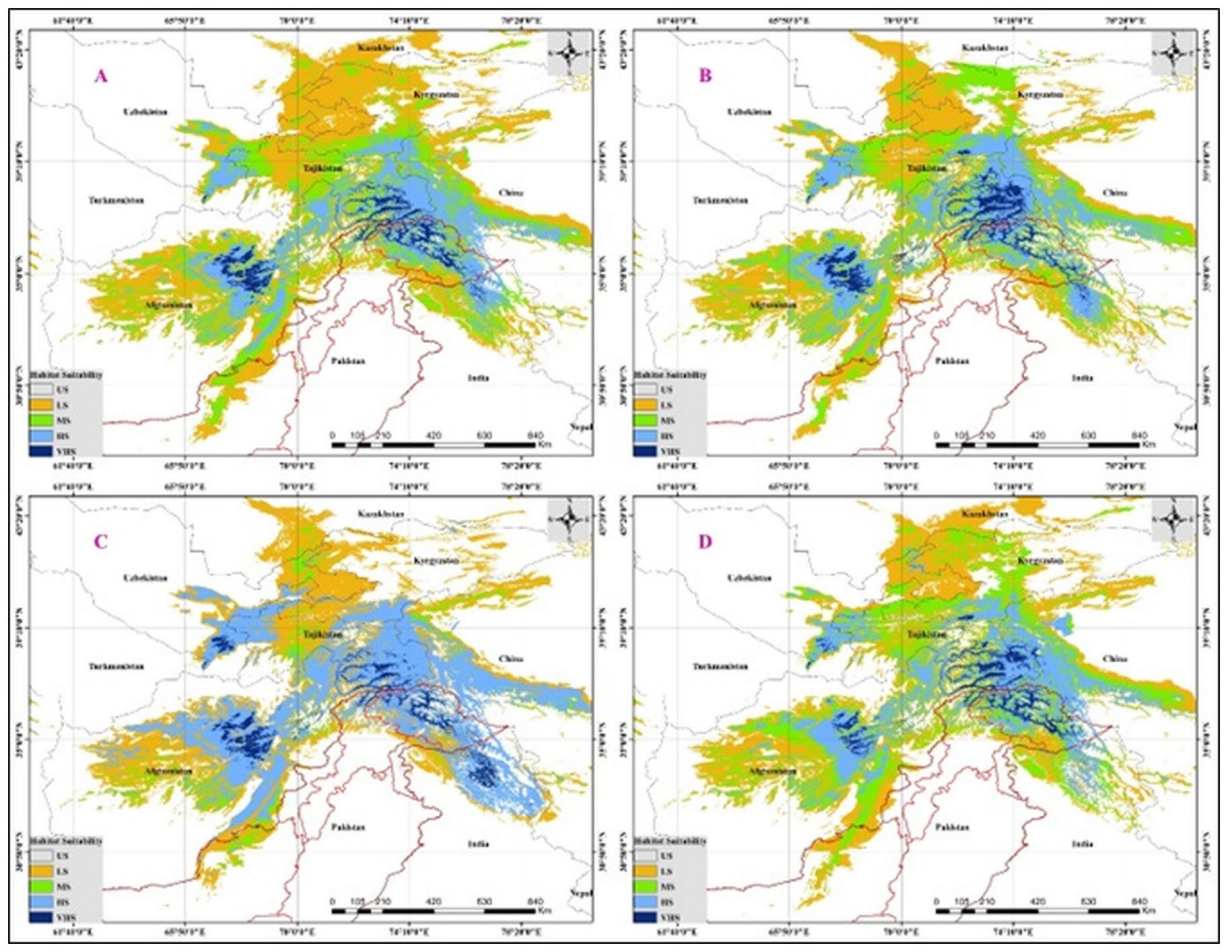
Predicted habitat suitability of *E. intermedia* under future climate change scenarios. Panels (A) and (B) show the projected distribution under the SSP2‐4.5 scenario for the 2050s and 2070s, respectively, representing a moderate emissions pathway. Panels (C) and (D) illustrate the projected distribution under the SSP5‐8.5 scenario for the 2050s and 2070s, representing a high‐emissions pathway. Habitat suitability is categorized into unsuitable (US, gray), low suitability (LS, green), moderate suitability (MS, yellow), high suitability (HS, blue), and very high suitability (VHS, dark blue).

The future distribution of *E. intermedia* was modeled under two climate change scenarios: SSP2‐4.5 (a moderate emissions pathway) and SSP5‐8.5 (a high‐emissions pathway) for both the 2050s and 2070s (Figure 7). The analysis indicates significant shifts in habitat suitability in response to changing climatic conditions, with notable differences between the two scenarios. Under the SSP2‐4.5 scenario in the 2050s, the model predicts a moderate contraction of highly suitable habitats (VHS) in the current core distribution areas, particularly in Afghanistan and northern Pakistan. However, suitable habitats expand slightly towards the northeast into parts of China and Kyrgyzstan. This suggests a potential shift in the range due to increasing temperatures and changing precipitation patterns, with some areas in the lower elevation zones becoming less suitable (shift from MS/HS to LS/US). By the 2070s, the SSP2‐4.5 scenario shows a more pronounced reduction in very HS areas, particularly in the western parts of the range (Afghanistan and western Pakistan). High and very HS zones are predicted to shrink, while areas of low to MS expand into previously US zones, especially towards the eastern parts of the distribution range (Tajikistan and China). This suggests a gradual eastward and upward migration of the species' suitable habitats. Under the more extreme SSP5‐8.5 scenario in the 2050s, the model projects a substantial reduction in the extent of VHS habitats. The core habitats in the Hindu Kush and Karakoram regions show signs of fragmentation, with a noticeable decrease in highly suitable areas. Conversely, new suitable habitats emerge at higher altitudes and further north, indicating a potential shift in the species' range as it tracks cooler temperatures at higher elevations and latitudes. By the 2070s under the SSP5‐8.5 scenario, the model predicts a drastic shift in the distribution of *E. intermedia* . VHS zones are largely confined to high‐altitude regions in the Himalayas and northern ranges of Pakistan and China. The western and southern parts of the range experience a marked decline in suitability, likely due to increased temperature stress and altered precipitation patterns. The expansion of US and LS areas highlights the potential risk of habitat loss under this high‐emissions scenario. Predictions under both SSP2‐4.5 and SSP5‐8.5 scenarios indicate a shift in the range of *E. intermedia* towards higher latitudes and elevations, accompanied by marked reductions in habitat suitability in the southern and western areas of its current range. The more severe SSP5‐8.5 scenario forecasts greater habitat loss and increased fragmentation, underscoring the species' heightened susceptibility to intense climate change impacts.

## 10 Discussion

The ENM developed for *E. intermedia* demonstrated high predictive accuracy. The strong model fit can be attributed to the inclusion of both bioclimatic and edaphic factors, reflecting the multifaceted ecological preferences of *E. intermedia* . Comparatively, previous studies on closely related species, such as *Ephedra sinica* and *Ephedra distachya* , reported similar predictive performance when utilizing bioclimatic variables in ENMs (He et al. 2021; Zhang et al. 2022; Li et al. 2024; Xu et al. 2024). However, the inclusion of edaphic variables (e.g., soil pH and nitrogen content) in this study provided a more comprehensive understanding of habitat suitability, underscoring the importance of integrating soil‐related factors in niche modeling for arid and semi‐arid‐adapted species like *E. intermedia* .

The analysis highlighted several key bioclimatic variables that significantly influenced the predicted distribution of *E. intermedia* . Notably, bio07 and bio04 emerged as critical predictors. The preference for moderate temperature seasonality and a specific annual temperature range suggests that *E. intermedia* is adapted to environments with marked seasonal temperature variations, which is consistent with its distribution in mountainous and semi‐arid regions. Previous studies have reported similar findings, where temperature seasonality was a primary determinant of habitat suitability for other *Ephedra* species (Guo, Gao, et al. 2023; Guo, He, et al. 2023; Zhang et al. 2024). The strong response to temperature‐related variables aligns with the physiological adaptations of *E. intermedia* to cope with temperature extremes (Tokuda et al. 2024). This suggests that the species' distribution is strongly constrained by temperature fluctuations, a pattern that is consistent across the other Gymnosperms (Subedi et al. 2020; Li et al. 2021). The response to bio15 further supports this interpretation. *E. intermedia* showed a higher suitability in regions with moderate precipitation seasonality, reflecting its adaptation to environments where water availability fluctuates seasonally. This finding is corroborated by previous niche modeling studies on desert and semi‐desert plants, which emphasize the importance of seasonal precipitation in shaping the distribution of drought‐tolerant species (Butterfield and Briggs 2011; Halmy et al. 2019; Herrando‐Moraira et al. 2020). The response curves indicated a strong positive relationship between habitat suitability and moderately alkaline soils (pH 5–15). This aligns with field observations and ecological studies that have noted the prevalence of *E. intermedia* in regions with alkaline or calcareous soils (Asensi et al. 2007; Rabizadeh et al. 2018). Alkaline soils typically have higher nutrient availability, which may explain the observed preference for these soil types, as the species is adapted to exploit nutrient‐poor but alkaline environments (Jobbagy and Jackson 2001; Adcock et al. 2007). The positive response to soil nitrogen content, a key environmental factor representing soil fertility, indicates that *E. intermedia* favors moderately fertile soils. Such soils may provide essential nutrients to support its growth, particularly in nutrient‐limited arid environments. Positive biotic interactions, including facilitation, may enhance the establishment and survival of *E. intermedia* by improving resource availability and reducing environmental stress in challenging conditions (Jäschke et al. 2020). This finding is consistent with previous research on nitrogen uptake strategies in desert plants, where moderate nitrogen levels were found to enhance plant performance without exacerbating competition with co‐occurring species (Zhuang et al. 2020). The response curve for elevation exhibited a bimodal distribution, indicating that *E. intermedia* is most suited to mid‐elevations (2000–3500 m). This elevational range corresponds to montane ecosystems, where the species is commonly found (Li et al. 2024). The preference for mid‐elevation habitats may be linked to favorable microclimatic conditions (Zhang et al. 2024; Xu et al. 2024).

The projected distribution of *E. intermedia* under future climate scenarios suggests significant range shifts, with a tendency for northward and upward migration of suitable habitats. Under both SSP2‐4.5 and SSP5‐8.5 scenarios, the model predicts a contraction of VHS areas, particularly in the southern and western parts of the current range. This finding aligns with broader trends observed across plant species in arid and mountainous regions, where warming temperatures and altered precipitation patterns drive species to track their climatic niches towards higher elevations and latitudes (Randin et al. 2009; Koo and Park 2022; Martínez et al. 2012). The upward and northward shift in the predicted range of *Ephedra intermedia* can be attributed to its sensitivity to temperature changes, as indicated by the strong response to bio04 and bio07. The species appears to be adapted to environments with moderate seasonal temperature fluctuations and a specific temperature range, conditions that are increasingly restricted to higher elevations under warming scenarios (Liu et al. 2009; La Sorte and Jetz 2010; Rumpf et al. 2018). As global temperatures rise, the suitable thermal niche of *E. intermedia* is expected to migrate upward, reflecting a broader trend observed in high‐altitude flora (Shaheen et al. 2023; Lin et al. 2023). This pattern of range shifts is consistent with the “escalator to extinction” hypothesis, which suggests that montane species may face increased risk of habitat loss as they are forced into progressively smaller and fragmented high‐elevation refugia (Zu et al. 2021; Waheed et al. 2024).

The SSP5‐8.5 scenario projects a substantial reduction in the extent of highly suitable habitats for *E. intermedia* . This contraction is most pronounced in the low‐elevation and southern parts of the range, where increased temperature stress and changes in precipitation regimes may exceed the physiological tolerances of the species (Marifatul et al. 2024; Waheed et al. 2024a). Similar findings have been reported for other arid‐adapted plants, where niche contraction under high‐emission scenarios is driven by the exacerbation of temperature extremes and reduced water availability (Waheed et al. 2023; Waheed and Arshad 2024; Arshad, Haq, et al. 2024; Arshad, Shoaib, et al. 2024). The potential for range contraction and habitat fragmentation under the SSP5‐8.5 scenario highlights the vulnerability of *E. intermedia* to climate change. The species' preference for specific temperature and precipitation conditions suggests that its realized niche may be relatively narrow, making it more susceptible to changes in climatic conditions (Wallingford et al. 2020; Atwater and Barney 2021). As temperatures rise and precipitation patterns become more erratic, the ecological niche of *E. intermedia* may shift towards areas that can still offer the combination of moderate temperature seasonality and drier conditions preferred by the species.

The predicted range shifts for *E. intermedia* are consistent with findings from previous studies on range dynamics of arid and montane plants under climate change (Rather et al. 2021; Du et al. 2021). For example, research on alpine plant species in the European Alps and the Qilian Mountains, China, reported similar upward and northward shifts as species track their climatic niches (Dagnino et al. 2020; Du et al. 2021). The phenomenon of range shifts in response to climate warming has been well documented across taxa, highlighting a global trend of biotic responses to increasing temperatures (Peng et al. 2021; Tiwari et al. 2024). In the context of the *Ephedra* genus, studies on *Ephedra sinica* and *Ephedra distachya* also indicated a tendency for upward range shifts under future climate scenarios (He et al. 2021; Zhang et al. 2022; Li et al. 2024). However, the inclusion of edaphic variables in this study provided additional understanding, suggesting that soil characteristics may play a critical role in modulating the species' response to climate change. This highlights the importance of considering multiple environmental dimensions in ENM to capture the full complexity of species' habitat preferences (Pshegusov et al. 2022; Arshad et al. 2022).

## 11 Implications for Conservation

The results of this study carry significant implications for the conservation efforts of *E. intermedia* , a species of medicinal and ecological significance. The predicted contraction of suitable habitats under high‐emission scenarios suggests that the species may face a substantial risk of population decline and local extirpation, particularly in the western parts of its range (Afghanistan and Pakistan). Conservation efforts should prioritize the protection of high‐elevation refugia and consider the potential for assisted migration to facilitate the species' adaptation to shifting climate conditions. Furthermore, the strong influence of edaphic factors on habitat suitability underscores the need to integrate soil characteristics into conservation planning. Protecting areas with alkaline and moderate nitrogen levels could enhance the resilience of *E. intermedia* populations, as these sites may offer critical microhabitats for the species under changing environmental conditions.

## 12 Limitations and Future Research Directions

While the model demonstrated strong predictive performance, several limitations should be acknowledged. The reliance on current bioclimatic data may not fully capture the microhabitat variations that influence the distribution of *E. intermedia* , particularly in heterogeneous mountain landscapes. Additionally, the model does not account for biotic interactions, such as competition and herbivory, which could influence the realized niche of the species. Future studies could integrate fine‐scale environmental data and biotic factors to enhance the predictive accuracy of ENMs for this and other similar species.

## 13 Conclusion

This study identifies the key ecological factors influencing the distribution of *E. intermedia* and predicts significant range shifts under future climate scenarios. The findings highlight the species' vulnerability to climate change and emphasize the importance of targeted conservation strategies. Protecting high‐altitude refugia and maintaining habitat connectivity are crucial to mitigating the impacts of environmental changes on *E. intermedia* . By providing actionable insights, this research contributes to the development of evidence‐based approaches for conserving arid and semi‐arid ecosystems, ensuring the persistence of ecologically significant species like *E. intermedia* in the face of global change.

## Author Contributions

**Muhammad Waheed:** conceptualization (equal), formal analysis (equal), investigation (equal), methodology (equal), validation (equal), visualization (equal), writing – original draft (equal), writing – review and editing (equal). **Fahim Arshad:** data curation (equal), resources (equal), supervision (equal), validation (equal), writing – review and editing (equal). **Sehrish Sadia:** data curation (equal), investigation (equal), writing – review and editing (equal). **Beatrice Ambo Fonge:** data curation (equal), validation (equal), writing – review and editing (equal). **Abeer Al‐Andal:** funding acquisition (equal), resources (equal), writing – review and editing (equal). **Asma Jabeen:** investigation (equal), project administration (equal), writing – review and editing (equal). **Shalom Dilshad:** data curation (equal), visualization (equal), writing – review and editing (equal).

## Conflicts of Interest

The authors declare no conflicts of interest.

## Supporting information


**Table S1.** Geographic distribution of Ephedra intermedia occurrence records used in this study. The table includes latitude (Lat) and longitude (Lon) coordinates for each recorded location.

**Table S2.** Environmental predictors from the studied locality are used in the MaxEnt species distribution model (SDM) for *Ephedra intermedia*.

## Data Availability

The data that support the findings of this study are available as a Supporting Information to this manuscript.
