# Supplementary material for: From Ecological Niche to Conservation Planning; Climate‐Driven Range Dynamics of Ephedra intermedia in Central Asia
Source: Ecol Evol. 2025 Mar 16;15(3):e71127. doi: 10.1002/ece3.71127 (PMC11911025; doi:10.1002/ece3.71127)
Supplement: Supplementary file 2 — Table S2. Environmental predictors from the studied locality are used in the MaxEnt species distribution model (SDM) for Ephedra intermedia. [file ECE3-15-e71127-s002.docx]

**Table S2.** Environmental predictors from the studied locality are used in the MaxEnt species distribution model (SDM) for *Ephedra intermedia*.

| Name of Variable & Description | Code | Unit | Resolution | Database |
| --- | --- | --- | --- | --- |
| Annual Mean Temperature | bio1 | °C | 30 arc s | WorldClim |
| Mean Diurnal Range | bio2 | °C | 30 arc s | WorldClim |
| Isothermality (Bio2/Bio7) (×100) | bio3 | Percent | 30 arc s | WorldClim |
| Temperature Seasonality (sd ×100) | bio4 | °C | 30 arc s | WorldClim |
| Max. Temperature of Warmest Month | bio5 | °C | 30 arc s | WorldClim |
| Min. Temperature of Coldest Month | bio6 | °C | 30 arc s | WorldClim |
| Temperature Annual Range | bio7 | °C | 30 arc s | WorldClim |
| Mean Temperature of Wettest Quarter | bio8 | °C | 30 arc s | WorldClim |
| Mean Temperature of Driest Quarter | bio9 | °C | 30 arc s | WorldClim |
| Mean Temperature of Warmest Quarter | bio10 | °C | 30 arc s | WorldClim |
| Mean Temperature of Coldest Quarter | bio11 | °C | 30 arc s | WorldClim |
| Annual Precipitation | bio12 | Mm | 30 arc s | WorldClim |
| Precipitation of Wettest Month | bio13 | Mm | 30 arc s | WorldClim |
| Precipitation of Driest Month | bio14 | Mm | 30 arc s | WorldClim |
| Precipitation Seasonality (CV) | bio15 | Percent | 30 arc s | WorldClim |
| Precipitation of Wettest Quarter | bio16 | Mm | 30 arc s | WorldClim |
| Precipitation of Driest Quarter | bio17 | Mm | 30 arc s | WorldClim |
| Precipitation of Warmest Quarter | bio18 | Mm | 30 arc s | WorldClim |
| Precipitation of Coldest Quarter | bio19 | Mm | 30 arc s | WorldClim |
| Bulk Density | bdod | cg/cm3 | 30 arc s | SoilGrids |
| Cations Exchange Capacity (pH: 7) | cec | mmol(c)/kg | 30 arc s | SoilGrids |
| Volumetric fraction of coarse fragments (>2 mm) | cfvo | cm3/dm3 | 30 arc s | SoilGrids |
| Clay Contents | clay | g/kg | 30 arc s | SoilGrids |
| Total Nitrogen | nitrogen | cg/kg | 30 arc s | SoilGrids |
| Organic Carbon Density | ocd | hg/dm3 | 30 arc s | SoilGrids |
| Soil pH × 10 | phh2o | Nil | 30 arc s | SoilGrids |
| Sand Contents | sand | g/kg | 30 arc s | SoilGrids |
| Silt Contents | silt | g/kg | 30 arc s | SoilGrids |
| Soil Organic Carbon | soc | dg/kg | 30 arc s | SoilGrids |
| Land cover | LC | Nil | 30 arc s | <http://www-modis.bu.edu/landcover> |
| Population density | PD | Nil | 30 arc s | http://www.ornl.gov/sci/landscan |
